# Microstructure and Performance of a Porous Polymer Membrane with a Copper Nano-Layer Using Vapor-Induced Phase Separation Combined with Magnetron Sputtering

**DOI:** 10.3390/polym9100524

**Published:** 2017-10-18

**Authors:** Nana Li, Yuanjing Fu, Qingchen Lu, Changfa Xiao

**Affiliations:** State Key Laboratory of Separation Membranes and Membrane Processes/National Center for International Joint Research on Separation Membranes, College of Textile, Tianjin Polytechnic University, Tianjin 300387, China; 1531015364@stu.tjpu.edu.cn (Y.F.); luqingchen@stu.tjpu.edu.cn (Q.L.); cfxiao@tjpu.edu.cn (C.X.)

**Keywords:** nano-layer, porous, membranes, sputtering, antibacterial

## Abstract

Antibacterial metalized poly(vinylidene fluoride) (PVDF) porous membranes with a nano-layer were obtained via the method of vapor-induced phase separation combined with magnetron sputtering of copper. Magnetron sputtering has such advantages as high deposition rates, low substrate temperatures, and good adhesion of films on substrates. The influence brought by deposition time on the microstructure, hydrophobic property, copper distribution state, anti-biofouling, and permeation separation performance was investigated via atomic force microscopy (AFM), field emission scanning electron microscopy (FESEM), energy-dispersive X-ray (EDX) spectrometry, contact angle measurements, and capillary flow porometry, along with the porosity, water flux, protein solution flux, rejection rate, water flux recovery rate, and antibacterial property. The results showed that copper particles formed island-type deposits on the membrane surface and were embedded into cross-section pores near the surface owning to the interconnection of pores. Subsequently, the water flux and protein solution flux declined, but the rejection rate and water flux recovery rate increased. Meanwhile, Cu-coated PVDF membranes exhibited an excellent antibacterial ability.

## 1. Introduction

The pollution problem encountered by poly(vinylidene fluoride) (PVDF) membranes affects their permeation flux and service life [1]. To effectively improve the antifouling property, nanoparticles are deposited on the PVDF membrane surface using dipping [2,3], sol-gel [4], chemical grafting [5], chemical vapor deposition [6], self-assembly [7], and atomic layer deposition [8]. Compared with these methods, magnetron sputtering has such advantages as high deposition rate, low substrate temperature, strong adhesion to the substrate, and scalability to large areas. This method has been widely used in industrial coatings for architectural glass, integrated circuits, and flexible substrates [9,10].

Magnetron sputtering has attracted significant attention in the membrane separation field, especially for water treatment membranes. Compared with pure membranes, membranes that are modified by sputtering a composite nano-layer showed better electrochemical and antifouling properties [11,12]. Vihodceva [13] reported a new approach using the magnetron sputtering to improve the fouling resistance of a reverse osmosis membrane. Bergamasco [14] applied a plasma treatment to modify a microfiltration cellulose acetate membrane with TiO_2_ and found that the modified membranes were able to not only remove color from raw water, but also present better results than membranes regarding membrane fouling and chlorine removal.

Previous studies have focused on the composite nano-layer, which provides the membrane with functional properties, such as physicochemical characteristics, as well as electrical and natural antibacterial properties. In this work, copper was used as a sputtering target material to modify the membrane pore structure. The influences of the copper distribution state on the membrane surface and internal pore structure were further studied. Specifically, the pores of the membrane surface and the subcortical structure can be embellished rather than directly blocked by copper particles embedded into the membrane cross-section. In addition, Cu-coated PVDF membranes exhibited an excellent antibacterial ability. This study contributes to the application of magnetron sputtering for surface modification of membranes. It is believed that metalized membranes can present excellent practical applications in the field of water treatment.

## 2. Experimental Methods and Materials

### 2.1. Materials

PVDF powders (W1300) were purchased from Kureha Chemical Industrial Co., Ltd. (Tokyo, Japan) with a melting temperature of 170 °C. *N*,*N*-dimethylformamide (DMF, analytical reagent) was obtained from Tianjin Kemiou Chemical Reagent Co., Ltd. (Tianjin, China). Bovine serum albumin (BSA, analytical reagent) was supplied by Beijing Aoboxing Universeen Bio-tech Co., Ltd. (Tianjin, China) with a weight-average molecular weight of 66,000 g/mol. Sodium chloride was purchased from Tianjin Fengchuan Chemical Reagent Technologies Co., Ltd. (Tianjin, China). Peptone and agar were supplied by Tianjin Yingbo Biochemical Reagent Co., Ltd. (Tianjin, China) and Chinese Pharmaceutical Group Shanghai Chemical Reagent Company, respectively. The copper target was supplied by Beijing Beiyi Innovation Vacuum Technology Co., Ltd. (Beijing, China) with a purity of 99.9%, a diameter of 10.3 cm and a thickness of 6 mm. Deionized water was used throughout the experiment.

### 2.2. Preparation of the PVDF Porous Membrane

Virgin PVDF membranes were obtained using the vapor induced phase separation (VIPS) method [15]. The casting solution, composed of 25 wt % PVDF and 75 wt % DMF, has been stirred at 50 °C for 4 h. The resulting solution was transferred into a vacuum drying oven at 50 °C to remove all bubbles. The solution was subsequently casted on a 500 μm thick glass plate. After being immediately placed in water vapor at 50 °C and 80 ± 2% relative humidity for 20 min, the solution films have been immersed in water for 48 h to remove residual solvent. Finally, the PVDF membranes were put in a freeze dryer (FD-1-50, Boyikang Co., Ltd., Beijing, China) for 24 h to obtain dry membranes.

### 2.3. Preparation of Copper-Metalized PVDF Membranes

Metalized PVDF membranes were prepared by a high vacuum magnetron sputtering system (GP450i, Beiyi Technology Co., Ltd., Beijing, China), with copper as the target and high purity Ar (99.9 at.%) as the environmental gas. The reaction conditions were as follows: substrate temperature of 25 °C, vacuum level below 10^−5^ mbar, and power of 40 W. Prior to the deposition of copper, the Cu target was pre-sputtered in an argon atmosphere for 30 min [16]. The deposition rate was varied to alter the membrane thickness, while the other parameters were fixed. The average deposition rate of Cu was 18 nm/min, and the maximum deposition rate was up to 25 nm/min, which was determined via several experiments; we performed our experiment using this rate. The Cu-metalized PVDF membranes with different deposition times of 5 min, 15 min, 30 min, and 45 min were labeled M5, M15, M30 and M45, respectively. The virgin PVDF membrane was designated as M0.

### 2.4. Characterization of the Membranes

The roughness and morphology of the membrane surface were observed through atomic force microscopy (AFM, Tap300Al, Benyuan Nano Instrument Co., Ltd., Beijing, China) and field emission scanning electron microscopy (FESEM), respectively. The chemical elemental composition of a small local area was determined by an energy dispersive X-ray (EDX, Nova Nano230, FEI Co., Haarlem, The Netherlands) micro-analyzer. The hydrophobic property was tested with 5 μL droplets at room temperature using a contact angle goniometer. 

The porosity (*ε*) of the membranes was measured according to the gravimetric method and was calculated using the following Equations (1) and (2) [17]:

(1) $$\varepsilon =\frac{M_{W}-M_{D}}{\rho_{w}A\sigma }$$

(2) $$A=\frac{1}{4}\pi d^{2}$$

where *M*_W_ is the wet weight, *M*_D_ is the dry weight (g), *ρ*_w_ is the density of *n*-butyl alcohol (0.810 g/cm^3^), *A* is the effective area (cm^2^), *δ* is the thickness of the wet membrane (cm), and *d* is the average diameter (cm).

The maximum pore diameter and pore diameter distribution were observed by the gas permeation method. The maximum pore diameter (*r*) can be defined by the Laplace Equation (3) [18]:

(3) $$r=\frac{2\sigma \cos\theta }{\Delta P}$$

where *σ* is the coefficient of the surface tension of the wetting fluid, *θ* is the contact angle between the wetting fluid and the membrane, and Δ*P* is the operating pressure when the first normal bubble appears. 

The dry flow (*F*_D_) and wet flow (*F*_W_) were measured. The filter flow (*F*) can be measured through Equation (4), and the pore diameter distribution (*B*) was defined by Equation (5) [19]:

(4) $$F(\%)=\frac{F_{W}}{F_{D}}\times 100\%$$

(5) $$B(\%)=\frac{F_{C}-F_{P}}{D_{P}-D_{C}}\times 100\%$$

where *D*_C_ is the permeation flux of the membrane, *D*_P_ is the pore diameter of the previous operating pressure, *F*_C_ is the filter flow of the current operating pressure and *F*_P_ is the filter flow of the previous operating pressure.

All experiments were performed based on the dead-end filtration system model. The permeate flux (*J*) was calculated using Equation (6):

(6) $$J=\frac{V}{A\cdot \Delta t}$$

where *J* is the permeation flux of the membrane (L·m^−2^·h^−1^), *V* is the volume of permeation (L), *A* is the effective area of the membrane (m^2^), and Δ*t* is the testing time (h). 

The BSA solution flux (*J*_BAS_) was measured by Equation (5). The recovery flux of the membrane (*J*_R_) was measured in pure water and calculated by Equation (5). The water flux recovery rate (*G*_WF_) was calculated by Equation (7), and the BSA rejection rate (*R*_BSA_) was measured using Equation (8) [20]:

(7) $$G_{\mathrm{WF}}(\%)=\frac{J_{W}-J_{R}}{J_{W}}\times 100\%$$

(8) $$R_{\mathrm{BSA}}(\%)=\left(1-\frac{C_{P}}{C_{F}}\right)\times 100\%$$

where *C*_P_ is the protein concentration of the permeation solution and *C*_F_ is the protein concentration of the feed solution.

Several tests were performed to characterize the antibacterial properties of the modified membranes.

The antibacterial property of the Cu-coated PVDF membranes was investigated by *Escherichia coli* (*E. coli*) via the shake flask method. The method was developed by Dow Corning Co. (Milland, MI, USA) and is now often used to evaluate the antimicrobial activity of samples that are treated with non-released reagents. *E. coli* was cultivated in a broth medium (containing 5 g/L yeast extract, 8 g/L peptone, 5 g/L sodium chloride) at 37 °C with shaking at 100 rpm for 24 h. Meanwhile, all membranes (2 g) were cut into pieces and sterilized with ultraviolet radiation and 75% alcohol. Then all membranes were immersed in 50 mL of 0.9% saline in a 250 mL sterile Erlenmeyer flask. Afterwards, 0.1 mL of bacterial suspension was pipetted into the flask, which was shaken at 100 rpm for 18 h. Finally, triplicate solid agar plates were coated by 0.1 mL of the suspension. The sealed plates were incubated at 37 °C for 24 h after being sealed to cause almost every viable bacterium to develop into a bacterial colony. The antibacterial properties of the prepared membranes were expressed via a bacterial colony count. The blank (without any membranes) and control (Cu-coated membranes with a different sputtering power) groups were also tested according to the above procedures. 

### 2.5. List of Abbreviations and Symbols

Poly(vinylidene fluoride) (PVDF); *N*,*N*-dimethylformamide (DMF); Bovine serum albumin (BSA); Vapor induced phase separation (VIPS); Atomic force microscopy (AFM); Field emission scanning electron microscopy (FESEM); Energy dispersive X-ray (EDX); Porosity of membranes (*ε*); Weight of wet membrane (*M*_W_); Weight of dry membrane (*M*_D_); Density of *n*-butyl alcohol (*ρ*_W_), Thickness of wet membrane (*δ*); Average diameter of the membrane(d); Maximum pore diameter (*r*); Coefficient of surface tension of the wetting fluid (*σ*), Contact angle (θ); Operating pressure when the first bubble appears (Δ*P*); Dry flow (*F*_D_); Wet flow (*F*_W_); Filter flow (*F*); Pore diameter distribution (*B*); Pore diameter corresponding to the current operating pressure (*D*_C_); Pore diameter corresponding to the previous operating pressure (*D*_P_); Filter flow corresponding to the current operating pressure (*F*_C_); Filter flow corresponding to the previous operating pressure (*F*_P_); Pure water flux (*J*_W_); Permeate flux of the membrane (*J*), Volume of the permeate (*V*), Effective area of the membrane (*A*); Testing time (Δ*t*); BSA solution flux (*J*_BAS_); Recovery flux of the membrane (*J*_R_); Water flux recovery rate (*G*_WF_); BSA rejection rate (*R*_BSA_); Concentration of protein in the permeate solution (*C*_P_); Concentration of the feed (*F*); Average roughness (Sa); Root mean square roughness (Sp); Mean particle size (Sq); and surface vertical height (Z).

## 3. Results and Discussion

### 3.1. Surface Morphology and Hydrophobic Property

**Figure 1:**
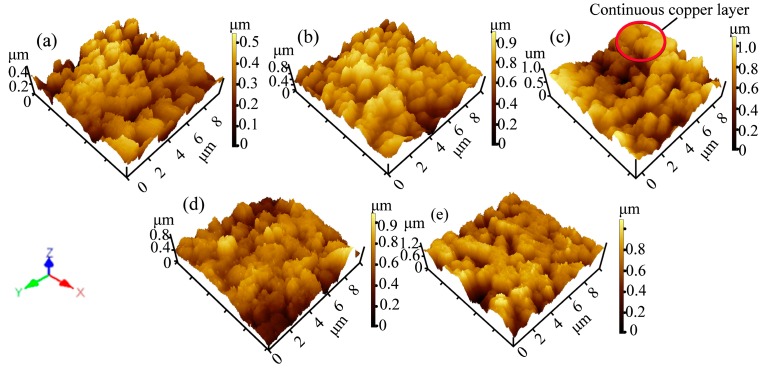
AFM images of the metalized membranes (10 μm × 10 μm) with different sputtering times: (**a**) M0; (**b**) M5; (**c**) M15; (**d**) M30; and (**e**) M45.

The three-dimensional AFM images can be observed in Figure 1. The roughness parameters and membrane surface particle diameters (Table 1) were obtained through AFM analysis software. The M0 surface showed less roughness with small PVDF particles (Figure 1a and Table 1). The mean diameter of the sputtered particles and surface roughness were increased during the initial sputtering time (Table 1). On the PVDF membrane surface, the copper particles formed island-shaped clusters at the beginning of sputtering (Figure 1b), and grew to large particles with sputtering. Meanwhile, some continuous layers appeared on the membrane surface because of the deposition of copper particles at the sunken sites between the islands, as shown in Figure 1c. The membrane surface roughness parameters were fully realized after 15 min of sputtering (Table 1). The surface roughness decreased as the islands grew, and then became closer. Finally, it became a relatively continuous layer. However the mean particle diameter of the membrane surface increased with further sputtering (Figure 1d,e and Table 1). The film formation mechanism has a direct influence on the film structure and optical-switching properties [21]. It is essential to understand the mechanism of film growth, which consists of particle–particle and particle–substrate interactions [22]. Firstly, the sputtered particles affected the nucleation activation energies and substrate environment, and the particles continued to diffuse on the membrane surface preferentially clustering on the weaker part of the substrate surface. In this state, the membrane surface was relatively rough. Then, the crystalline agglomeration was arranged and interconnected with each other to appear as an island-like pattern. When the sputtering time was extended, the continuous layer was obtained as copper particles deposited on the network cracks. As a result, the thickness of the comparatively smooth membrane increased.

**Figure 2:**
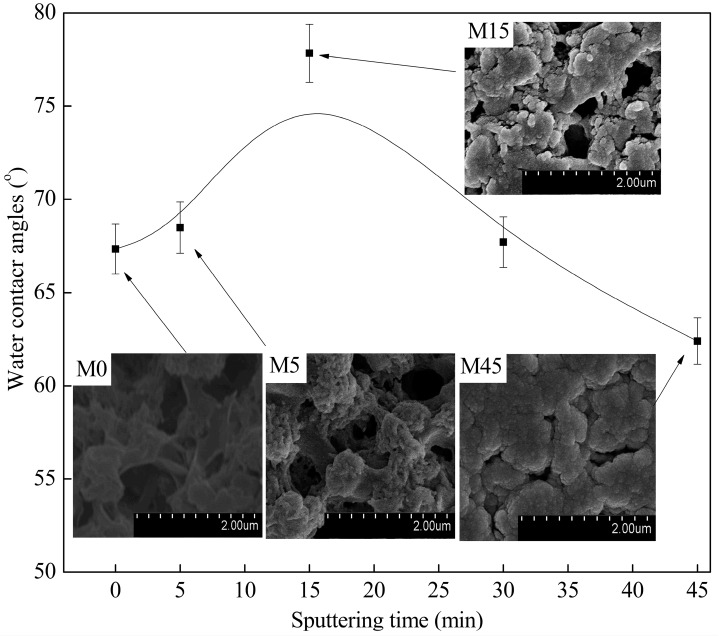
Water contact angles and surface FESEM images of the metalized membranes with different sputtering times.

It was clearly shown that the virgin membrane has a porous skin layer, which consists of the PVDF spherical particles due to the slow double diffusion between the solvent and non-solvent during the VIPS process (Figure 2 (M0)). Moreover, the water contact angle increased at the beginning, and then decreased with sputtering, which is associated with the surface roughness (Figure 1). First, the micro-nano structure, which was similar to the lotus leaf surface, was constructed by copper nanoparticles and PVDF spherical micro-particles during the initial stage of sputtering (less than 15 min, Figure 2 (M5, M15)) [23]. Subsequently, the surface roughness only increased over a reasonable range of sputtering time (less than 15 min), which was good for improving the hydrophobic property. Moreover, if the sputtering time was more than 15 min, the membrane surface became smoother (as can be seen in Table 1) due to the interconnection of the copper particles, which caused the water contact angle to decrease. This result demonstrated that the variation in the roughness of the membrane really had significant influence on the hydrophobic property of the membranes. The copper content on the surface mainly affects the surface energy.

### 3.2. The Distribution State of Copper

**Figure 3:**
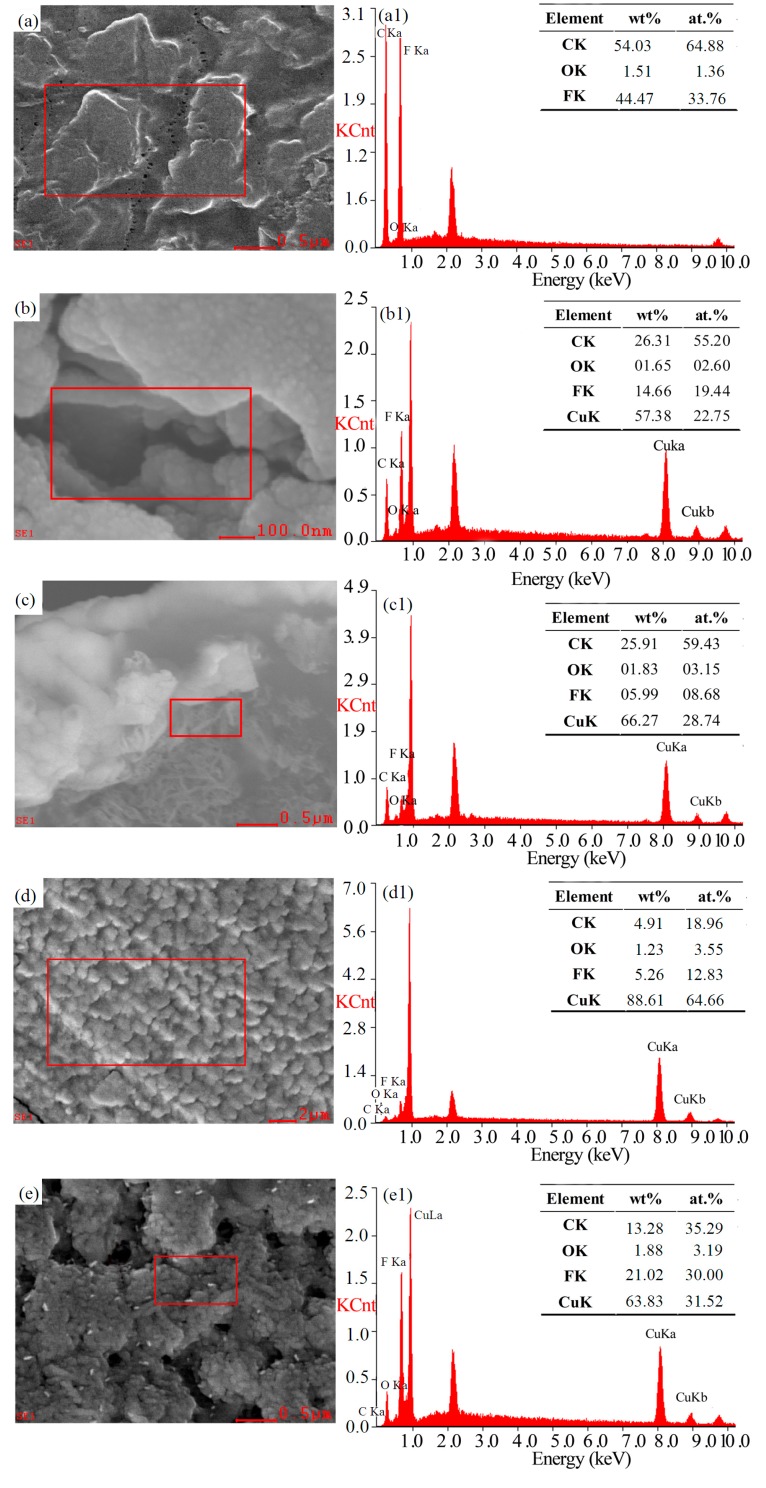
Chemical elemental composition measured using EDX for M0 (**a**,**a1**), M15 (**b**,**b1**,**c**,**c1**), and M45 (**d**,**d1**,**e**,**e1**): (**a**,**b**,**d**) surface morphology, (**a1**,**b1**,**d1**) elements on the surface, (**c**,**e**) cross-section morphology, and (**c1**,**e1**) elements at the cross-section near the surface.

The elemental composition of the surface area (the square in Figure 3a,b,d) and cross-section area (the square in Figure 3c,e) of M0, M15, and M45 was analyzed through EDX. The figure shows that the mass fraction and atomic percent of the F element on M0 surface are 44.47 wt % and 33.76 at.%, respectively (Figure 3a1). When the sputtering time reached 15 min, the mass fraction and atomic percent of the F element were 14.66 wt % and 19.44 at.% respectively. The F element on membrane surface was more than that in cross-section near the surface. Simultaneously, the mass fraction and atomic percent of the copper element were 57.38 wt % and 22.75 at.% in the surface pores, and 66.27 wt % and 28.74 at.% in the cross-section of pores near the surface, respectively (Figure 3b1,c1). These results demonstrated that the virgin PVDF membrane was not completely covered by copper. In general, the results showed that the copper particles were not only deposited on the membrane surface, but also embedded into the cross-section pores near the surface because of the interconnection of lager pores near the membrane surface (Figure 3b). The ratio of copper was higher in the cross-section pores near the surface due to the preferential growth of copper at the defect position during the initial stage of sputtering [24]. However, as the sputtering time was prolonged, a continuous copper layer was formed on the membrane surface (Figure 3d). The copper particles cannot enter into cross-section of membrane. Thereby, the content of copper in cross-section near the surface was almost constant, even though the content of copper on the surface increased as the sputtering time increased (Figure 3d1,e1).

### 3.3. Pore Structure and Permeation Separation Performances

**Figure 4:**
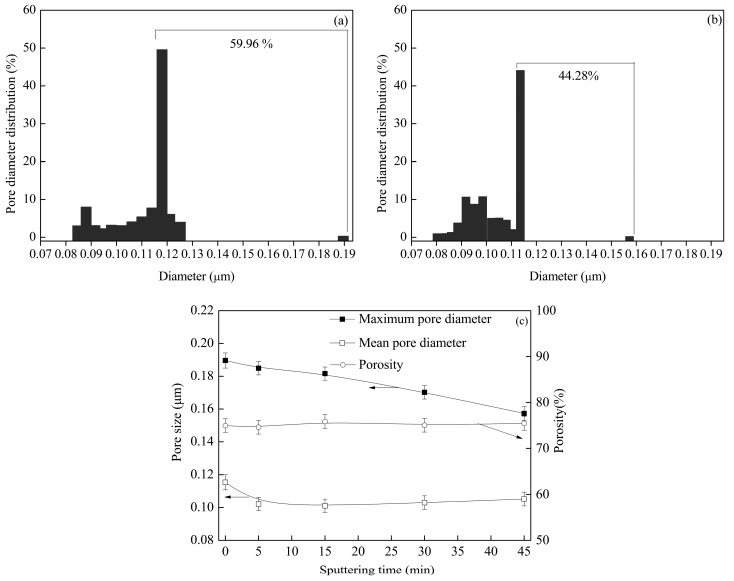
Pore diameter distribution of (**a**) M0 and (**b**) M45 and the (**c**) porosity, maximum pore diameter, and mean pore diameter of the metalized membranes with different sputtering times.

The distribution, mean value, maximum pore diameter, and porosity are shown in Figure 4. The results showed that no clear differences were observed in the porosity and mean pore diameter for the virgin PVDF membrane and the metalized membrane. This was attributed to almost all pores existing in the interior of membrane, and they were not affected by magnetron sputtering. However, the large pores mainly existed on surface. The percent of larger pores decreased from 59.96% to 44.28% with sputtering due to deposition of copper particles on the large pores, which are located on the membrane surface and at the cross-section near the surface. This conclusion is consistent with the distribution state of the copper on the metalized membranes (Figure 3). Similarly, the decrease in pore number and pore size with sputtering can be observed from the FESEM images in Figure 2. 

**Figure 5:**
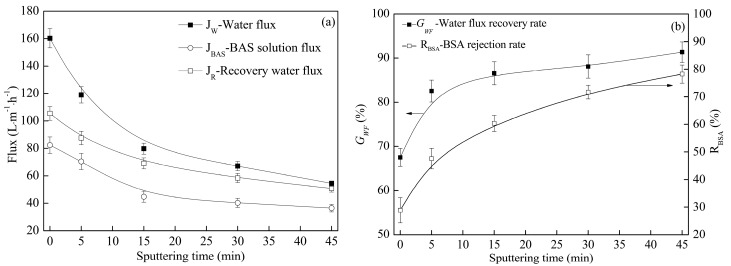
Permeation separation performance (including pure water flux, recovery flux, BSA solution flux, water flux recovery rate, and BSA rejection rate) of the metalized membranes with different sputtering times.

When the sputtering time was extended, the values of *J*_W_ (pure water flux), *J*_R_ (recovery flux of the membrane) and *J*_BAS_ (BSA solution flux) decreased, but the values of *G*_WF_ (water flux recovery rate) and *R*_BAS_ (BSA rejection rate) increased, as shown in Figure 5. The permeation separation performances were primarily controlled by the hydrophobic property and the pore structure. At the beginning of sputtering, the improvement in the hydrophobic property (Figure 2) caused the values of *J*_W_, *J*_R_, and *J*_BAS_ to decrease. The hydrophobic property became weaker as the sputtering time was prolonged over 15 min, and the copper particles were deposited on the large pores, which are located on the membrane surface and at the cross-section near the surface. It caused both the porosity and mean pore diameter to decrease (Figure 4). The rejection rate is inversely proportional to the pore size. As a result the values of *J*_W_, *J*_R_ and *J*_BAS_ decreased whereas the value of *R*_BAS_ increased. As the deposition time stretched from 0 min to 45 min, which effectively improved the fouling resistance of the modified membranes, the water flux recovery rate grew from 68% to 90%, and the BSA rejection rate rose from 55% to 79% (Figure 5). Moreover, M0 had a lower *G*_WF_ value because its low surface energy facilitated the adsorption of organic pollutants, which may block the pores. Furthermore, the copper layer prevented the membrane surface from taking in organic pollutants effectively, causing the *G*_WF_ value of the metalized membranes to increase. The result also illustrates the sharp increasing trend of *G*_WF_ when the sputtering time is less than 15 min, which is attributed to the deposition of continuous copper layers on the membrane surface.

### 3.4. Antibacterial Activity

**Figure 6:**
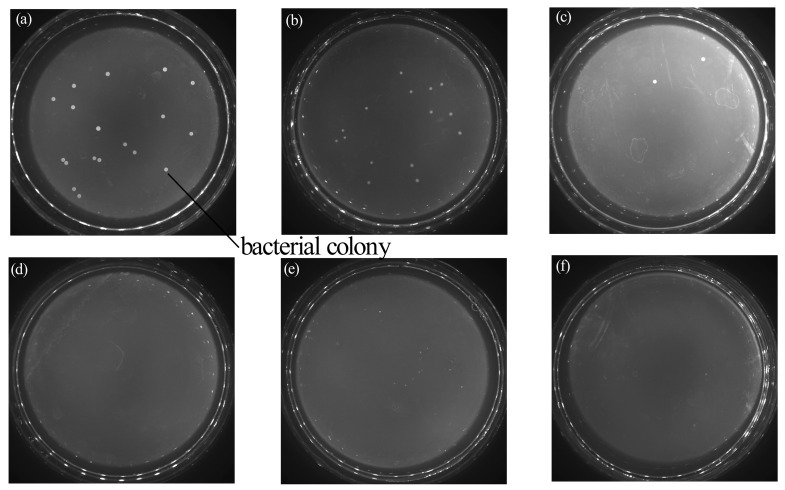
Media photos that correspond to the sample membranes with antibacterial properties under different sputtering times. (**a**) Blank control group; (**b**) virgin membranes; (**c**) 5 min; (**d**) 15 min; (**e**) 30 min; and (**f**) 45 min.

The antibacterial properties of the neat PVDF membrane and Cu-coated PVDF membrane were investigated against *E. coli* via the shake flask method, as shown in Figure 6. It was clearly observed that almost no bacterial colonies were present in the Petri dish (Figure 6e–f) while photos of the control group and the bare membrane showed a homogeneous film of *E. coli* bacteria (Figure 6a,b). In addition, Cu-coated membranes at 15 min and 30 min showed no colonies, while the composite membranes prepared at 5 min had several colonies. These phenomena indicate that the Cu-coated membranes exhibited an efficient antibacterial activity and the antibacterial efficiency can be enhanced by lengthening the coating time. This result was primarily thanks to the presence of Cu particles on the natural antibacterial property. The mechanism of the antibacterial activity of the Cu-coated PVDF membranes can be interpreted as follows. Copper ions, which are generated via the oxidation reaction of copper deposited on the membrane, can absorb bacterial cells onto the surface and kill them by generating reactive hydroxyl radicals. Therefore, some irreparable damages might be induced, such as protein oxidation, cleavage of DNA and RNA molecules, and cell membrane damage, due to lipid peroxidation [25,26].

## 4. Conclusions

In this work, the porous PVDF membranes with a copper nano-layer were built by the method of vapor-induced phase separation and magnetron sputtering. The distribution state of copper and its effect on the microstructure, hydrophobic property, antibacterial property, and permeation separation performances were discussed. The conclusions were as follows: Firstly, it can be displayed that copper grew like islands on the membrane surface. Actually, the surface roughness raised and arrived at a maximum when the sputtering time was 15 min, and then decreased with further sputtering. As the hydrophilic property has a positive correlation with surface roughness, the water contact angle also increased firstly, and then decreased with sputtering. M15 had the worst hydrophilic property. Secondly, copper particles not only deposited on membrane surface, but also imbedded into the cross-section pores near surface due to interconnection of larger pores near the surface. The result of copper deposition on the pores diminished the number and diameter of the larger pores, rather than being directly blocked. Finally, the water flux and protein solution flux gradually went down. However, the rejection rate and water flux recovery rate ascended with sputtering. Meanwhile, good antibacterial activity of the Cu-coated PVDF membranes was obtained thanks to the bactericidal property of the copper ions. This study contributes to the application of magnetron sputtering for membrane preparation. 

## Figures and Tables

**Table 1:** Mean diameter and surface roughness of the metalized membranes with different sputtering times.

Membrane	Mean Diameter (μm)	Surface Roughness (μm)		
	S_p_	S_a_	S_q_	Z
M0	0	0.071	0.0897	0.546
M5	0.067	0.118	0.121	0.903
M15	0.138	0.133	0.142	0.914
M30	0.175	0.130	0.129	1.101
M45	0.193	0.090	0.105	1.430
